# Supplementary material for: Resolving the full spectrum of human genome variation using Linked-Reads
Source: Genome Res. 2019 Apr;29(4):635–45. doi: 10.1101/gr.234443.118 (PMC6442396; doi:10.1101/gr.234443.118)
Supplement: Supplemental Material [file supp_gr.234443.118_Supplemental_longranger-2.2.zip › longranger-2.2.tar.bz2!/lr2.2-public/lib/stan/cmdstan/stan/doc/api/html/arg__bfgs_8hpp_source.html]

Stan: src/stan/gm/arguments/arg\_bfgs.hpp Source File


|  |  |
| --- | --- |
|  | Stan  2.5.0  probability, sampling & optimization |


- Main Page
- Related Pages
- Namespaces
- Classes
- Files

- File List
- File Members

All Classes Namespaces Files Functions Variables Typedefs Enumerations Enumerator Friends Macros Pages

- src
- stan
- gm
- arguments

arg\_bfgs.hpp

Go to the documentation of this file.

1 #ifndef STAN\_\_GM\_\_ARGUMENTS\_\_BFGS\_\_HPP

2 #define STAN\_\_GM\_\_ARGUMENTS\_\_BFGS\_\_HPP

3

4 #include <stan/gm/arguments/categorical\_argument.hpp>

5

6 #include <stan/gm/arguments/arg\_init\_alpha.hpp>

7 #include <stan/gm/arguments/arg\_tolerance.hpp>

8

9 namespace stan {

10

11  namespace gm {

12

13  class arg\_bfgs: public categorical\_argument {

14

15  public:

16

17  arg\_bfgs() {

18

19  \_name = "bfgs";

20  \_description = "BFGS with linesearch";

21

22  \_subarguments.push\_back(new arg\_init\_alpha());

23  \_subarguments.push\_back(new arg\_tolerance("tol\_obj","Convergence tolerance on absolute changes in objective function value",1e-12));

24  \_subarguments.push\_back(new arg\_tolerance("tol\_rel\_obj","Convergence tolerance on relative changes in objective function value",1e+4));

25  \_subarguments.push\_back(new arg\_tolerance("tol\_grad","Convergence tolerance on the norm of the gradient",1e-8));

26  \_subarguments.push\_back(new arg\_tolerance("tol\_rel\_grad","Convergence tolerance on the relative norm of the gradient",1e+7));

27  \_subarguments.push\_back(new arg\_tolerance("tol\_param","Convergence tolerance on changes in parameter value",1e-8));

28

29  }

30

31  };

32

33  } // gm

34

35 } // stan

36

37 #endif

38

stan::gm::arg\_tolerance

**Definition:** arg\_tolerance.hpp:12

stan::gm::arg\_init\_alpha

**Definition:** arg\_init\_alpha.hpp:10

categorical\_argument.hpp

arg\_init\_alpha.hpp

stan::gm::categorical\_argument

**Definition:** categorical\_argument.hpp:11

stan::gm::categorical\_argument::\_subarguments

std::vector< argument \* > \_subarguments

**Definition:** categorical\_argument.hpp:153

stan::gm::arg\_bfgs::arg\_bfgs

arg\_bfgs()

**Definition:** arg\_bfgs.hpp:17

arg\_tolerance.hpp

stan::gm::argument::\_name

std::string \_name

**Definition:** argument.hpp:76

stan::gm::arg\_bfgs

**Definition:** arg\_bfgs.hpp:13

stan::math::e

double e()

Return the base of the natural logarithm.

**Definition:** constants.hpp:86

stan::gm::argument::\_description

std::string \_description

**Definition:** argument.hpp:77


---

[ Stan Home Page ]

*© 2011–2014,
Stan Development Team.*
